# STRENGTHENING BRAIN HEALTH AND DEMENTIA RESOURCES FOR OUR RELATIVES OF NORTHWEST TRIBES

**DOI:** 10.1093/geroni/igae098.1228

**Published:** 2024-12-31

**Authors:** Chandra Wilson

**Affiliations:** Northwest Portland Area Indian Health Board, Portland, Oregon, United States

## Abstract

The Northwest Tribal Elders Project (NTEP) will manage the resources and provide space for the current tribal dementia relatives to gather and share intertribal stories of our traditional ways of health and wellness to address Alzheimer’s disease and related dementia (ADRD) among I/T/U (Indian Health Services, Tribal and Urban Indian) clinics. The aim of the project is to provide technical assistance, training, and resources within our 43-member tribes’ elder programs and tribal programs supporting older adults. NTEP collaborates with all 43-member tribes and provides ADRD resources that may be tailored to the health and wellness practices within each tribal community. A tribal coalition will provide input on the implementation of our ADRD Strategic plan to promote risk reduction and intervention priorities, early detection screenings and assessments and education and awareness of ADRD. Through this relationship powerful and impactful things are going to happen, the hope is there will be an increase of discussions happening with our member tribes to discuss ADRD, mild cognitive impairment, memory care and caregiving. The tribe’s input on training and technical assistance needs is the medicine for our loved ones whom are being cared for.

